# Supplementary material for: Measurement of the axial vector form factor from antineutrino–proton scattering
Source: Nature. 2023 Feb 1;614(7946):48–53. doi: 10.1038/s41586-022-05478-3 (PMC9892001; doi:10.1038/s41586-022-05478-3)
Supplement: Supplementary file 1 — Supplementary Tables 1–7. [file 41586_2022_5478_MOESM1_ESM.pdf]

nature portfolio

https://doi.org/10.1038/s41586-022-05478-3

Supplementary information

Measurement of the axial vector form factor from antineutrino–proton scattering

In the format provided by the authors and unedited

Supplementary Material.

Supplementary Table 1 Measured events and predicted backgrounds. Measured events, post-fit Monte Carlo background prediction, measured signal, and the statistical uncertainties after background subtraction, in each Q^2 bin. Only bins 3-17 are statistically significant and reported in the cross-section.

Bin	$Q^2_{\rm low}$	$Q^2_{\rm up}$	Measured Event	Pred. BG	Signal	Stat. Err
1	0	0.00625	0	0	0	-
2	0.00625	0.0125	1	0	1	1
3	0.0125	0.025	11	3.3	7.7	3.6
4	0.025	0.0375	18	8.3	9.7	4.6
5	0.0375	0.05	31	22.2	8.8	7.0
6	0.05	0.1	328	182	146	23
7	0.1	0.15	684	372.0	312	32.9
8	0.15	0.2	1011	523.7	487.3	39.1
9	0.2	0.3	2507	1401	1106	65
10	0.3	0.4	2485	1563	922	66
11	0.4	0.6	3908	2861	1047	84
12	0.6	0.8	2540	1919	621	69
13	0.8	1	1604	1262	342	55
14	1	1.2	1019	788.3	230.7	42.0
15	1.2	2	1494	1164	330	53
16	2	4	395	289	106	25
17	4	6	37	20.6	16.4	6.8
18	6	10	8	4.4	3.6	3.1

Supplementary Table 2 Protons on Target (POT) and Number of Hydrogen Atoms in the Fiducial Volume of the Detector.

POT	1.12e+21
Number of Hydrogen Atoms	2.61e+29

Supplementary Table 3 Measured and fitted cross-sections. Binned cross-section (10^{-38}) with the statistical and systematic uncertainties. The predicted cross-section using the z-expansion fit with different combinations of ($k_{\max}, \lambda$) are shown after the data column, with the total uncertainty shown in the bracket.

Bin	Q^2_{low}	Q^2_{up}	$d\sigma/dQ^2(10^{-38})$	stat.	sys.	Fit(8, 0.13)	Fit(6, 0)
3	0.0125	0.025	3.06	1.82	0.47	1.90(5)	1.90(4)
4	0.025	0.0375	0.779	0.73	0.22	1.81(7)	1.81(6)
5	0.0375	0.05	0.596	0.547	0.196	1.73(9)	1.73(7)
6	0.05	0.1	1.25	0.353	0.195	1.55(0.11)	1.55(9)
7	0.1	0.15	1.13	0.179	0.153	1.31(0.12)	1.31(0.11)
8	0.15	0.2	0.962	0.114	0.124	1.13(0.12)	1.13(0.10)
9	0.2	0.3	0.979	0.0834	0.107	0.92(0.10)	0.92(9)
10	0.3	0.4	0.821	0.0765	0.0673	0.71(8)	0.71(8)
11	0.4	0.6	0.49	0.0525	0.0579	0.50(5)	0.50(5)
12	0.6	0.8	0.296	0.0455	0.0418	0.317(35)	0.32(4)
13	0.8	1.0	0.206	0.0387	0.0343	0.208(25)	0.209(25)
14	1.0	1.2	0.138	0.0297	0.0243	0.140(19)	0.141(19)
15	1.2	2.0	0.0561	0.0133	0.00835	0.063(12)	0.063(11)
16	2.0	4.0	0.0112	0.00525	0.00213	0.0096(32)	0.010(4)
17	4.0	6.0	0.00587	0.004	0.002	0.00057(34)	0.0006(5)

Supplementary Table 4 Z expansion fit parameters and results. The a_k parameters given without uncertainties are not free variables in the fit but set by the sum rule in Eq. 13.

$k_{\max}$	λ	r_A	$a_0, \ldots, a_{k_{\max}}$
8	0.13	0.73(17)	$-0.50, 1.50(0.31), -1.2(0.7), -0.1(1.9), 0.2(3.5), 0.46, -0.40, 0.15, -0.044$
6	0	0.72(20)	$-0.50, 1.50(0.33), -1.2(0.5), -0.13, 0.15, 0.42, -0.23$

Supplementary Table 5 Fit correlation matrices. The z-expansion fit correlation matrices for the free a_k in the fit, i.e., $a_1 \ldots a_4$ for $k_{\max} = 8$ and a_1, a_2 for $k_{\max} = 6$.

$k_{\max}$	λ	Correlation Matrix
8	0.13	$\begin{pmatrix} 1. & 0.012 & -0.93 & 0.52 \\ 0.012 & 1. & -0.32 & -0.78 \\ -0.93 & -0.32 & 1. & -0.27 \\ 0.52 & -0.78 & -0.27 & 1. \end{pmatrix}$
6	0	$\begin{pmatrix} 1. & -0.73 \\ -0.73 & 1. \end{pmatrix}$

Supplementary Table 6 Total covariance matrix of the measured cross-section

Bin($\times 10^{-80}$)	3	4	5	6	7	8	9	10	11	12	13	14	15	16	17
3	35400.	1220.	-1500.	-242.	311.	311.	128.	65.8	125.	52.9	49.1	47.4	0.899	-1.21	-3.52
4	1220.	5800.	2480.	27.4	-38.6	92.2	72.5	27.4	6.01	1.54	1.01	6.85	2.64	1.62	0.376
5	-1500.	2480.	3370.	784.	-11.7	63.2	76.5	46.8	37.5	29.	12.8	4.43	1.46	-0.217	0.237
6	-242.	27.4	784.	1630.	248.	84.1	117.	61.9	62.	45.5	20.6	5.59	-1.24	0.0773	-0.575
7	311.	-38.6	-11.7	248.	554.	222.	102.	53.4	53.6	37.2	17.	5.05	-1.87	0.111	-0.656
8	311.	92.2	63.2	84.1	222.	284.	128.	34.5	40.3	28.9	15.1	7.67	-1.04	0.211	-0.443
9	128.	72.5	76.5	117.	102.	128.	184.	59.	22.9	23.8	14.4	6.98	-0.02	0.382	0.135
10	65.8	27.4	46.8	61.9	53.4	34.5	59.	104.	27.4	12.4	10.6	7.12	1.92	0.278	0.495
11	125.	6.01	37.5	62.	53.6	40.3	22.9	27.4	61.	22.8	10.3	6.2	1.63	0.0879	0.0404
12	52.9	1.54	29.	45.5	37.2	28.9	23.8	12.4	22.8	38.2	15.8	4.67	0.966	0.102	0.0698
13	49.1	1.01	12.8	20.6	17.	15.1	14.4	10.6	10.3	15.8	26.7	12.5	0.927	-0.0491	0.0693
14	47.4	6.85	4.43	5.59	5.05	7.67	6.98	7.12	6.2	4.67	12.5	14.7	2.2	-0.247	0.0263
15	0.899	2.64	1.46	-1.24	-1.87	-1.04	-0.02	1.92	1.63	0.966	0.927	2.2	2.46	0.0602	0.0196
16	-1.21	1.62	-0.217	0.0773	0.111	0.211	0.382	0.278	0.0879	0.102	-0.0491	-0.247	0.0602	0.32	-0.00339
17	-3.52	0.376	0.237	-0.575	-0.656	-0.443	0.135	0.495	0.0404	0.0698	0.0693	0.0263	0.0196	-0.00339	0.194

Supplementary Table 7 Statistical only covariance matrix of the measured cross-section

Bin($\times 10^{-80}$)	3	4	5	6	7	8	9	10	11	12	13	14	15	16	17
3	28900.	966.	-1410.	-466.	50.9	19.1	-2.11	0.011	-0.0296	-0.0451	-0.022	-0.00704	0.00136	-0.000117	-0.0000439
4	966.	4640.	2160.	11.5	-86.2	3.8	4.95	-0.254	-0.0498	-0.00699	-0.0117	-0.00328	0.00173	0.0000244	-0.0000352
5	-1410.	2160.	2610.	484.	-122.	-19.3	6.47	0.585	-0.00713	-0.0124	-0.00844	-0.0052	-0.00102	0.00017	0.00000806
6	-466.	11.5	484.	1090.	-5.86	-71.7	-1.84	5.31	0.17	-0.129	0.0122	-0.00934	-0.0152	0.000653	0.00021
7	50.9	-86.2	-122.	-5.86	278.	41.1	-27.2	-2.29	1.98	0.3	-0.102	-0.045	0.00539	-0.00287	-0.000183
8	19.1	3.8	-19.3	-71.7	41.1	114.	6.83	-15.2	-0.111	0.848	0.189	0.0613	-0.0111	-0.00173	-0.0000753
9	-2.11	4.95	6.47	-1.84	-27.2	6.83	60.6	1.54	-7.08	-0.117	0.646	0.193	0.0219	-0.000453	-0.000791
10	0.011	-0.254	0.585	5.31	-2.29	-15.2	1.54	50.9	1.01	-5.12	-0.825	0.238	0.113	0.006	-0.0009
11	-0.0296	-0.0498	-0.00713	0.17	1.98	-0.111	-7.08	1.01	24.	0.68	-3.3	-1.15	0.0393	0.0246	0.00172
12	-0.0451	-0.00699	-0.0124	-0.129	0.3	0.848	-0.117	-5.12	0.68	18.1	2.91	-1.89	-0.588	0.0242	0.00983
13	-0.022	-0.0117	-0.00844	0.0122	-0.102	0.189	0.646	-0.825	-3.3	2.91	13.	4.16	-0.849	-0.106	0.0191
14	-0.00704	-0.00328	-0.0052	-0.00934	-0.045	0.0613	0.193	0.238	-1.15	-1.89	4.16	7.69	0.606	-0.255	-0.0138
15	0.00136	0.00173	-0.00102	-0.0152	0.00539	-0.0111	0.0219	0.113	0.0393	-0.588	-0.849	0.606	1.54	-0.0204	-0.0312
16	-0.000117	0.0000244	0.00017	0.000653	-0.00287	-0.00173	-0.000453	0.006	0.0246	0.0242	-0.106	-0.255	-0.0204	0.24	-0.00517
17	-0.0000439	-0.0000352	0.00000806	0.00021	-0.000183	-0.0000753	-0.000791	-0.0009	0.00172	0.00983	0.0191	-0.0138	-0.0312	-0.00517	0.139
